# The Third French Alzheimer Plan: analysis of the influence of a national public health initiative on scientific research productivity and impact

**DOI:** 10.1186/s13195-015-0144-z

**Published:** 2015-09-26

**Authors:** Nicole Haeffner-Cavaillon, Patrick Devos, Sylvie Ledoux, Joël Ménard

**Affiliations:** INSERM, Department of Scientific Evaluation, Bibliometric Unit, 101 Rue Tolbiac, F-75014 Paris, France; Department of Research, University of Lille, CHU Lille, EA2694, F-59000 Lille, France; Fondation Plan Alzheimer, F-75013 Paris, France; Faculté de médecine Paris-Descartes, 15 rue de l’Ecole de Médecine, Paris, France; Centre d’Investigations Cliniques 1418, F-75006 Paris, France

## Abstract

**Introduction:**

The Third National Alzheimer Plan (2008–2012) was a major public health initiative in France that included €200 million of funding for research in Alzheimer disease and related disorders (AD). The aim of this study was to document trends in French academic output in AD following the implementation of the plan.

**Methods:**

Academic output (i.e., number of original articles) and scientific impact (i.e., article citations) of French research in AD were obtained from the Web of Knowledge core collection database. Analyses compared the 5-year period immediately before (2004–2008) and after (2009–2013) initiation of the plan. Comparisons were made with stroke, Parkinson disease, AIDS, and diabetes in the 14 leading countries worldwide and regionally within France.

**Results:**

Worldwide production of original scientific articles between the periods 2004–2008 and 2009–2013 increased by 39 %, and that for AD increased by 46 %. China showed the largest increase and Japan the smallest. The absolute increase in French output on AD (54.6 %) was larger than that for stroke, Parkinson disease, AIDS, or diabetes. Globally, France had the third largest relative increase in output in AD (1.7-fold), behind only India (2.5-fold) and China (1.9-fold). There was a relative 2.3-fold increase in the proportion of French AD articles in the top 1 % globally most cited, larger than that for French articles on stroke, Parkinson disease, AIDS, or diabetes. At the national level, university hospitals participated in nearly 50 % of French AD publications. Analyses by geographical area demonstrated marked heterogeneity. We observed a strong correlation between level of funding and volume of output (*R*^2^ = 0.70), but not between funding and article impact (proportion of top 10 % globally cited articles; correlation *R*^2^ = 0.03).

**Conclusions:**

Our study provides evidence of a specific positive impact of the funding provided by the Third National Alzheimer Plan in nearly doubling the global academic scientific output and increasing by 2.3-fold the top 1 % globally cited articles of France in AD research. Our bibliometric analyses provide objective and transparent information for policy makers on the relationship between research funding and academic output.

**Electronic supplementary material:**

The online version of this article (doi:10.1186/s13195-015-0144-z) contains supplementary material, which is available to authorized users.

## Introduction

A national public health plan is a politically driven choice aimed at the mobilization of financial and human resources to reduce the burden of selected diseases. Since 2001, France, along with several other countries, has successively implemented three national plans against Alzheimer disease and related disorders (AD). The most recent and comprehensive was the Third National Alzheimer Plan, driven directly by the presidency of the French Republic from 2008 to 2013, in which research was included for the first time as one of the three major objectives of the plan: “to understand, to cure, to care” [1]. Funding for AD was shared between research (€200 million), medical care (€200 million), and medicosocial support (€1.2 billion). The research funding was allocated by three major bodies: the National Agency for Research (ANR; under the Ministry of Higher Education and Research), the Hospital Program for Clinical Research (PHRC; under the Ministry of Social Affairs and Health), and the Alzheimer Plan Foundation (created to implement the overall initiative). Whereas university hospitals (CHUs) preferentially benefit from PHRC funds, universities in partnership with national public research organizations (RPOs; e.g., Institut National de la Santé et de la Recherche Médicale (INSERM), Centre National de la Recherche Scientifique (CNRS), Commissariat à l'Energie Atomique (CEA), and higher education institutions) received funding mainly through ANR applications. Both research organizations were eligible for funding from the Alzheimer Plan Foundation.

Given the level of commitment required for a 5-year national public health plan, it is important to quantify for the scientists, sponsors, and contributors, in a transparent fashion, the scientific impact of the investment made. It is also necessary to review the appropriateness of resource allocation and to provide an objective basis for designing further research strategies at a regional and national level. With these objectives, we performed a bibliometric analysis assessing in parallel the impact of the Third National Alzheimer Plan on both French academic scientific output and the attribution of research funding [2], comparing the 5-year period immediately before the plan (2004–2008) with the 5 years after initiation of the plan (2009–2013). The relative contribution of France to the global literature on AD was assessed in terms of both the quantity (i.e., number of articles) and quality (i.e., representation in the top 1 % and top 10 % of globally cited publications) relative to four other major disease areas (stroke, Parkinson disease, HIV, and diabetes) and benchmarked by international comparisons.

## Methods

### The Alzheimer Plan Foundation

A non-profit private foundation was created in June 2008 by the French Ministry of High Education and Research to coordinate and implement the research measures of the Third National Alzheimer Plan. The Foundation connects multiple governmental funding sources, such as the ANR and the PHRC, to support research projects on basic, clinical, or social research and also plays the part of a think tank to encourage public and private financial support. Five major pharmaceutical companies operating in France (AstraZeneca, Merck Sharp & Dohme, Servier, Sanofi, and Ipsen) provided some financial support as a permanent guarantee. These private founders were members of the board of directors but had no input on the scientific options.

### Bibliometric analyses

To evaluate the international and national impact of the Third French Alzheimer Plan, bibliometric analyses were performed in July 2014 to examine the production of original scientific articles from France in comparison with other countries and diseases other than AD. The analyses compared the 5-year period immediately before the plan (2004–2008) with the 5 years after initiation of the plan (2009–2013) and were conducted using the Thomson Reuters Web of Knowledge core collection database. All analyses included only full articles in peer-reviewed journals; other forms of publication were excluded (i.e., proceedings papers, reviews, abstracts, obituaries, corrections, editorials).

#### Research output

Research output was defined as the number of articles published in journals covered by the Science Citation Index, the Social Science Citation Index, and the Arts and Humanities Citation Index in the Web of Science core collection database.

#### Comparisons with other countries and major disease areas

Research output was evaluated in AD and related neurodegenerative dementias in comparison with four other major comparator disease areas (stroke, Parkinson disease, HIV, and diabetes) by use of appropriate search terms in the topic search. The search included the title, abstract, keywords, and Thomson Reuters Keywords Plus fields. AD and related neurodegenerative dementia terms searchers were “Alzheimer” or “fronto-temporal dementia” or “semantic dementia” or “posterior cortical atrophy ” or “progressive aphasia” or “Body Lewy Body disease” or “beta amyloid” or “amyloid precursor” or “APP and processing” or “presenilin” or “tau protein.” Articles related to CADASIL, Parkinson, Creutzfeldt-Jakob, prion, a myotrophic Lateral Sclerosis, Huntington, multiple sclerosis, Niemann-Pick, Down syndrome, or Wilson disease were discarded because they were not supported by the Third National Alzheimer Plan. As Thomson Reuters KeyWords Plus consist of words and phrases harvested from the titles of the cited articles and are embedded in the topic search, we excluded articles containing the following terms: “epilepsy” or “autism” or “schizophrenia” or “bipolar glioma” or “carcinoma” or “stroke” or “cancer” or “child” or “fetal” or “infant” or “newborn” or “preterm” or “pediatric” or “prenatal” or “neonatal” or “infant” or “adolescent” or “stroke” or “trauma.”

HIV terms searched were “acquired immune deficiency syndrome” or “HIV.” Diabetes terms searched were “diabetes” or “diabetic.” The stroke term searched was “stroke.” For international and national comparative assessments, country and city information was obtained from the author field. Each publication was counted in full for every institution or country identified in the author affiliations; that is, our assumption was that each author, institution, and country in the listed affiliations made a non-negligible contribution.

#### Top 1 % and top 10 % publications

Globally highly cited publications were identified using Thomson Reuters Essential Science Indicators (ESI) thresholds, which select the top 1 % or top 10 % of articles by total citations in each annual cohort from each of the 22 ESI domains. Each article is assign to 1 of the 22 scientific domains (and only 1) based on the journal in which it appears. These indicators allow one to identify the “essential core” of journal articles within a research field. The title, abstract, and summary of each article were manually screened for the top 1 % globally cited articles for comparison of the top six European Union (EU) countries and the top 10 % globally cited articles for comparison of geographical areas in France.

#### Output increase

Output increase represents the absolute percentage increase in the output of publications by a given entity (i.e., a country or French region) between the periods 2004–2008 and 2009–2013.

#### Output relative increase

Output relative increase represents the percentage increase in the output of publications by a given entity (i.e., a country or French region) between the periods 2004–2008 and 2009–2013, divided by the overall percentage increase of the world (for analyses by country) or France (for analyses by French region) between those periods.

#### Relative citation impact

The citation count for each publication attributed to a field was divided by the mean citations for that field in the year of publication.

#### SIGAPS score

The SIGAPS system has been used in France since 2006 to assess the research contribution of all CHUs. It relies on a classification of the publications into five classes (A–E) based on the journal impact factor distribution in each research topic’s category as defined by the Journal Citation Report Science Edition (JCR 2013 edition). For each article, the SIGAPS score (from 1 to 32) is calculated based on the class of the journal in which the article was published and the position in the author list of the author affiliated with the CHU. When several researchers of one CHU coauthor the same article, it is counted once for the institution with the highest score [3].

#### Research field assessment

The research fields of articles comprised clinical investigation (including clinic cohorts), biology (including clinical chemistry, experimental models, biochemistry, experimental and cellular pharmacology), neuroimaging, epidemiology (population cohorts), genetics, and neuropathology. Research fields were allocated based on the journal (using the Thomson Reuters ESI domains), the article title, the content of the abstract, and the specialization of the main authors.

#### Geographical research areas in France

Twenty-nine geographical research areas were defined by a specific query using the corresponding French cities hosting universities and CHUs. The city-based queries included the suburb cities where affiliated hospitals, universities, and RPOs are located; for example, the Lyon research area included Lyon, Pierre-Bénite, Bron, and Villeurbanne. Analysis focused on the scientific performance of the major French research areas, including publications from RPOs, higher education institutes, universities, and CHUs with at least 50 articles dedicated to AD between 2004 and 2013. CHU output was obtained from the SIGAPS system referencing over 70,000 authors belonging to the 29 CHUs. For Paris, the research area was divided into the 13 Paris universities; for example, Paris 11 included Orsay, Gif-sur-Yvette, Saclay, Cachan, Châtenay-Malabry, Bures-sur-Yvettes, Villejuif, and Bicêtre. As CHUs of Paris are affiliated with Assistance Publique-Hôpitaux de Paris, hospital affiliations were corrected manually. Each hospital article was attributed to its university; for example, publications from Hôpital Pitié-Salpêtrière, Hôpital Charles-Foix, Hôpital Saint-Antoine, Hôpital Tenon, Hôpital Rothschild, and Hôpital Trousseau-La Roche-Guyon were attributed to the Paris 06 research area. For high-impact articles (top 10 %), the bibliometric outputs for the French geographical research areas were also screened manually by author affiliation. In some specific cases, top 10 % articles were attributed to specific regional research areas only if one author was in a leading position (i.e., corresponding author). Each top 10 % article was attributed to only one research area.

## Results

### Allocation of funding in the Third French Alzheimer Plan, 2008–2012

The Third French Alzheimer Plan included a total of €200 million dedicated to research, of which €45 million were already engaged through RPOs, €5–7 million for doctoral positions and staff positions through universities, €1 million (0.5 %) for the Alzheimer Plan Foundation management and €126 million distributed through grant applications. The Ministry of Health supported 50 clinical research projects through PHRCs (€24.7 million), €34.1 million was provided through the ANR, and €41.9 million was distributed through the Alzheimer Plan Foundation (Additional file 1: Figure S1a).

Grant applications for specific research projects were reviewed by committees of independent, largely non-French experts. The quality of the grant applications assessed by experts was the primary criterion for selection. There was minimal consideration given to allocation to specific fields of research, and no geographic targeting. Similar levels of funding support were allocated to clinical investigation (36.5 % of research funds) and to fundamental research (24.3 % for biology and 8.2 % for genetics). Almost 20 % of the funds were dedicated to imaging and technology and more than 10 % to social sciences and epidemiological studies (Additional file 1: Figure S1b).

### Regional funding and scientific output in Alzheimer disease in France

The impact of the Third French Alzheimer Plan on scientific output was assessed for the major French research areas (*n* = 29), including publications from universities, RPOs, higher education institutes, and CHUs. Production was assessed in terms of both volume (number of articles) and impact (proportion of papers in the top 10 % globally cited and the SIGAPS score for CHUs). For the period 2004–2008, five geographical research areas produced at least 100 articles related to AD (Paris 06, Toulouse, Paris 11, Lille, and Bordeaux). During the period 2009–2013, output increased in all geographical research areas and 9 produced at least 100 articles related to AD (Paris 06, Toulouse, Bordeaux, Montpellier, Paris 11, Lille, Paris 05, Lyon, and Marseille).

The global quantitative increase in scientific output in AD (+69.6 %) also appeared to yield an increase in impact, as measured by the number of French articles in the top 10 % globally cited: For 57.5 % of the top 10 % cited articles, a French author has a leading position. We observed an increase of 134 % (from 189 to 442) in the number of top 10 % articles between the periods 2004–2008 and 2009–2013. In 2004–2008, 5 research areas led (corresponding author) more than ten top 10 % articles, whereas in 2009–2013 a total of 13 research areas led more than ten top 10 % articles (Table 1). It is noteworthy that the percentage of top 10 % articles with a corresponding author was above 60 % for the Nice, Caen, and Lille research areas. The distribution of top 10 % cited articles by research field did not change markedly between the two time periods, but there were notable increases in the number of top 10 % articles in neuroimaging, genetics, and epidemiology, three fields that received almost 30 % of French Alzheimer Plan funding.

**Table 1:** Change in the number and proportion of articles in leadership of French research areas between periods 2004–2008 and 2009–2013

	2004–2008 top 10 % publications			2009–2013 top 10 % publications			Share of funding (%)
Research areas	Leadership, n	Global top 10 %, n	Leadership, %	Leadership, n	Global top 10 %, n	Leadership, %	2009–2013
Nice	2	6	33.3	17	25	68.0	5.2
Caen	13	16	81.3	20	30	66.7	2.1
Lille	10	15	66.7	36	56	64.3	10.6
Toulouse	16	21	76.2	46	80	57.5	6.4
Paris 07	9	13	69.2	12	21	57.1	4.5
Rouen	2	6	33.3	13	24	54.2	3.1
Paris 05	6	8	75.0	13	26	50.0	6.9
Angers	0	2	0.0	11	22	50.0	1.5
Bordeaux	15	24	62.5	23	51	45.1	10.5
Paris 06	23	34	67.6	28	63	44.4	14.1
Paris 11	7	17	41.2	18	46	39.1	3.5
Lyon	5	11	45.5	12	33	36.4	3.6
Montpellier	9	13	69.2	16	53	30.2	4.5

**Fig. 1:**
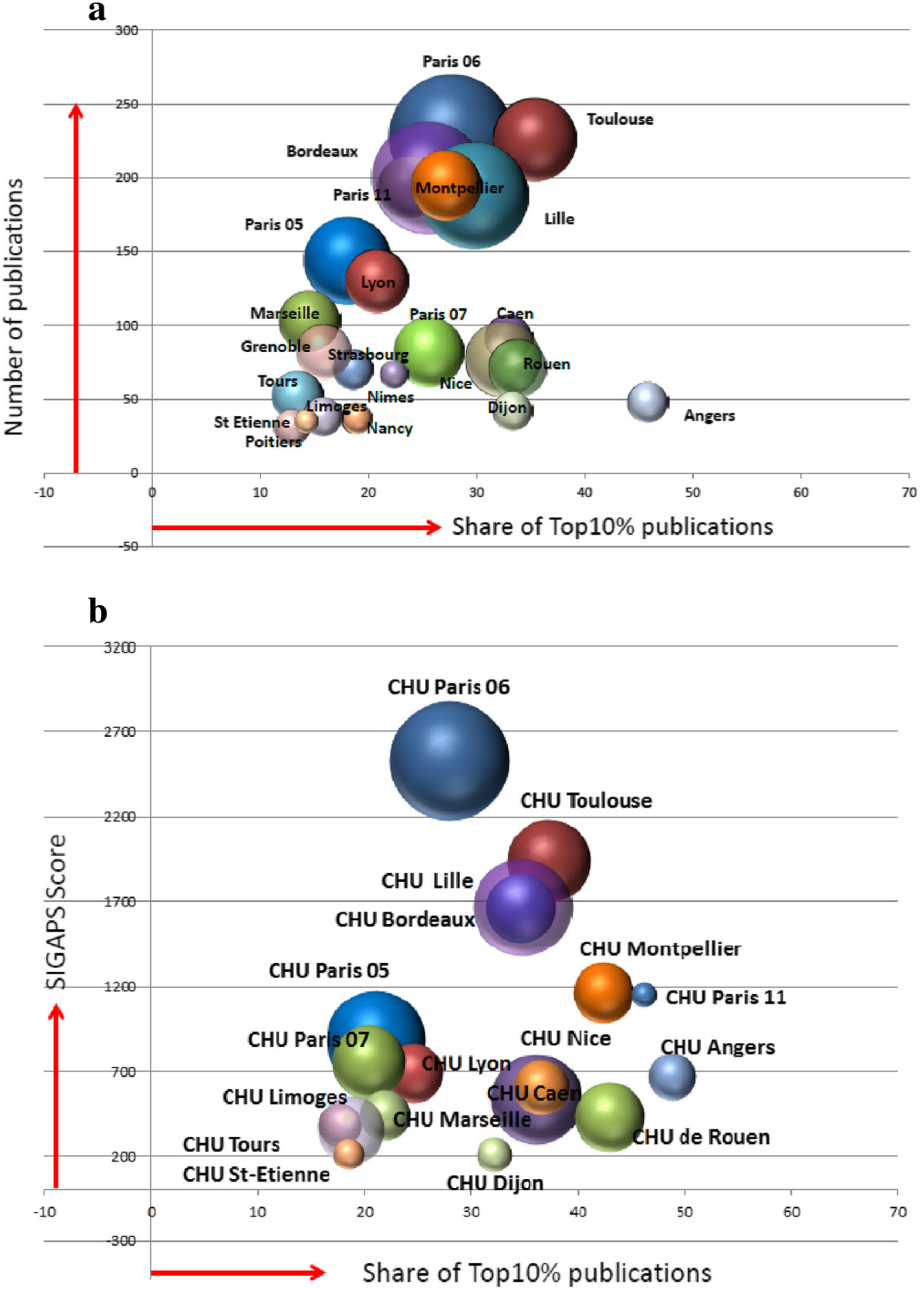
Relationship between level of funding received from the Third French Alzheimer Plan, the volume (number of articles/SIGAPS score), and impact (representation in top 10 % globally cited articles) of Alzheimer disease research in French geographical research areas and university hospitals (CHUs) in 2009–2013. **a** Relationship for each geographical research area of the total number of Alzheimer disease articles, the number of articles in the top 10 % globally cited, and the relative level of funding received from the Third French Alzheimer Plan (size of bubble). **b** Relationship for each CHU of the SIGAPS score for Alzheimer disease articles (a measure of research output that takes into account factors including journal impact factor and the position of the authors within the author list), the number of articles in the top 10 % globally cited, and the relative level of funding received from the Third French Alzheimer Plan (size of bubble)

Figure 1a shows, for the 2009–2013 period, the relationship between AD scientific output in terms of volume (number of articles) and impact (proportion of top 10 % globally cited articles, independent of author leadership), as well as the level of funding (size of the bubbles) in the major geographical research areas. Overall, there was a significant correlation (*R*^2^ = 0.70) between the level of financial support and the volume of scientific output (number of articles), but there was no correlation (*R*^2^ = 0.03) between the level of funding and the impact of scientific output (proportion of top 10 % articles). The latter result reflects the observation that several geographical areas (e.g., Angers. Dijon, Rouen, Caen, and Nice) delivered a high proportion of top 10 % cited articles despite relatively modest levels of funding.

Figure 1b shows the results of the analysis performed for CHUs using the SIGAPS author’s affiliation. Overall, the mean contribution of CHUs involved in AD research was 48 % of French publications. CHU involvement in AD research was variable within the geographical research areas. Five of the CHUs contributed to more than 50 % of the SIGAPS-indexed publications: hospitals in partnership with the University Paris 06, as well as the CHUs from Toulouse, Bordeaux, Lille, and Montpellier.

As with the Web of Science analysis, we observed a high correlation (*R*^2^ = 0.96) between the SIGAPS score and the volume of scientific output (number of articles) for CHUs, but there was no correlation (*R*^2^ = 0.19) between the SIGAPS score and the proportion of top 10 % articles.

### Volume of French scientific research output in Alzheimer disease

To evaluate if the increase of scientific research output in AD in France was specific and not due to a global increase in research activity, we compared the change in scientific output between the periods 2004–2008 and 2009–2013 in the most productive countries and compared output in AD with that in stroke, Parkinson disease, HIV, and diabetes.

**Fig. 2:**
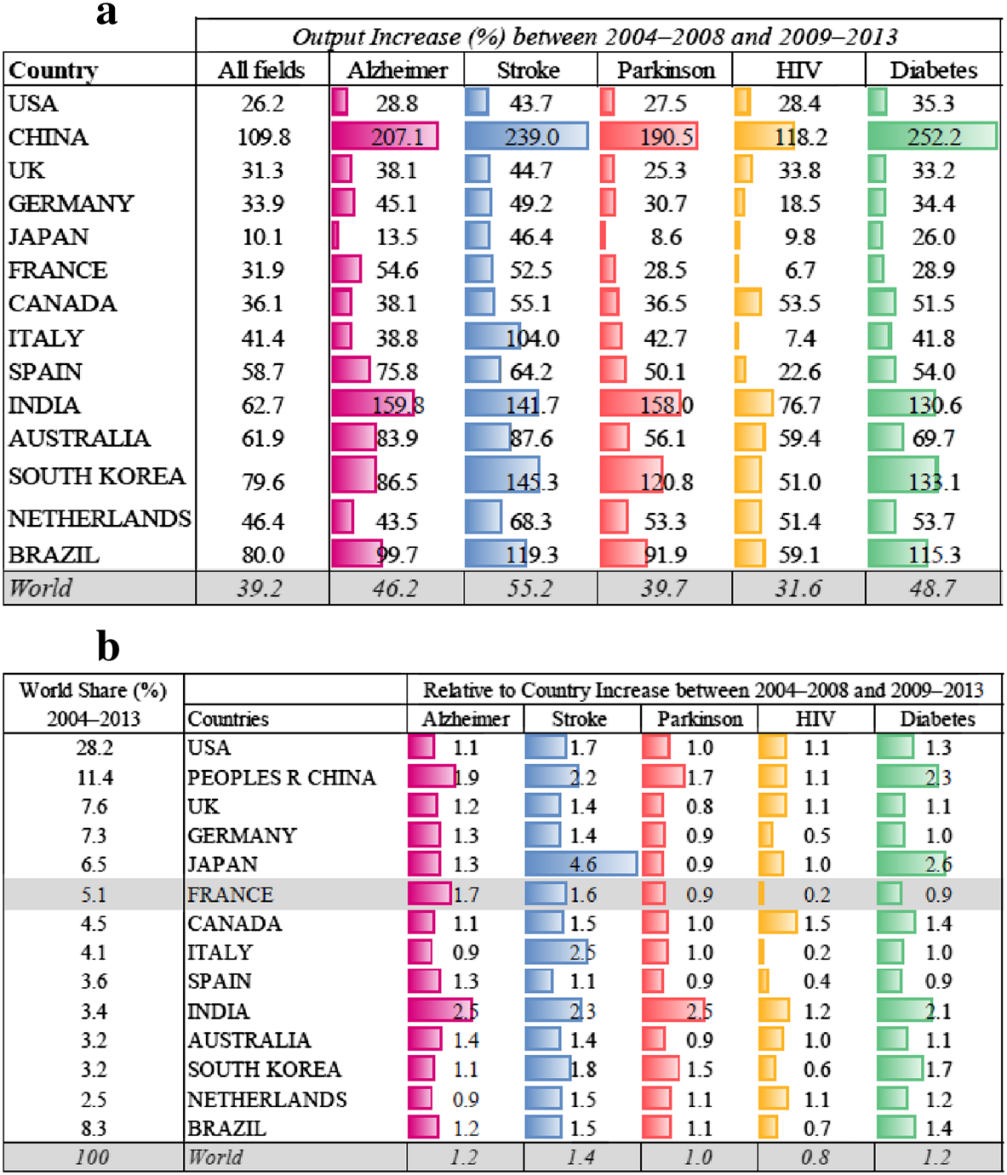
Increase in country publication output between the periods 2004–2008 and 2009–2013 for five major disease areas (Alzheimer disease, stroke, Parkinson disease, HIV, and diabetes) in absolute terms and relative to the overall output within each country. **a** Percentage increase in the number of publications overall and in each disease area between the periods 2004–2008 and 2009–2013. The top 14 countries worldwide are ranked according to their contribution during the period 2004–2008. **b** For each country, the fold increase in the proportion of publications in each disease area relative to the overall increase in the number of publications between the periods 2004–2008 and 2009–2013 is shown. The top 14 countries worldwide are ranked according to their contribution during the period 2004–2008

Figure 2a shows that global scientific output across all research fields in the Web of Science database increased in all countries, with an overall 39 % increase between the periods 2004–2008 and 2009–2013 (Table 2). The United States was by far the largest contributor of scientific articles in both periods, but its relative global contribution fell from 30 % to 27 % of total articles, reflecting the dramatic rise in the number of articles produced in countries such as China, India, South Korea, and Australia. France increased its output by 32 % between the two time periods, but its global share decreased from 5.3 % to 5.0 %. The global share for the United States, the United Kingdom, and other European countries declined as well. For AD, global scientific output increased by 46 % between the periods 2004–2008 and 2009–2013, which was greater than the increase for Parkinson disease (40 %) and HIV (32 %) but lower than the output observed for stroke (55 %) and diabetes (49 %). The absolute increase in scientific output for AD was higher than the global mean for China, India, Brazil, South Korea, Australia, Spain, and France.

**Table 2:** Number of published articles for the top 14 countries worldwide during the time periods 2004–2008, 2009–2013 and 2004–2013, across all disease areas and specifically in Alzheimer disease and the four major comparator disease states (stroke, Parkinson disease, HIV and diabetes)

	All			Alzheimer		Stroke		Parkinson		HIV		Diabetes	
	2004–2013	2004–2008	2009–2013	2004–2008	2009–2013	2004–2008	2009-2013	2004–2008	2009-2013	2004–2008	2009–2013	2004–2008	2009–2013
United States	2,949,083	1,303,555	1,645,528	11,260	14,499	11,561	16,615	6537	8335	20,976	26,938	25,006	33,842
China	1,188,127	383,509	804,618	1247	3830	1289	4370	665	1932	1577	3441	2413	8499
United Kingdom	795,735	343,455	451,080	2487	3435	3241	4689	1767	2214	4080	5461	5822	7752
Germany	757,711	323,922	433,789	2037	2956	2950	4400	1772	2316	1928	2285	4516	6069
Japan	675,315	321,389	353,926	2240	2543	2328	3408	1451	1576	1369	1503	5706	7192
France	537,568	231,875	305,893	1250	1932	1485	2264	997	1281	3123	3331	2890	3726
Canada	467,998	198,202	269,796	1448	1999	1974	3061	992	1354	1963	3014	3227	4889
Italy	424,650	175,930	248,720	1775	2463	1358	2770	1232	1758	2090	2244	3681	5219
Spain	375,748	145,233	230,515	1010	1776	1029	1690	817	1226	1811	2221	2185	3364
India	353,056	134,381	218,675	296	769	319	771	207	534	1201	2122	1512	3487
Australia	334,610	127,767	206,843	822	1512	1172	2199	510	796	1298	2069	2442	4144
South Korea	329,696	117,933	211,763	743	1386	892	2188	432	954	337	509	1588	3702
Netherlands	257,829	104,649	153,180	830	1191	1207	2031	531	814	1106	1675	2137	3285
Brazil	861,367	93,334	168,033	377	753	450	987	270	518	1273	2025	1384	2980
World	10,439,490	4,364,842	6,074,648	26,190	38,302	32,552	50,506	17,219	24,059	42,679	56,171	68,575	101,968

Figure 2b shows the relative change in scientific output between the periods 2004–2008 and 2009–2013 in each disease area in the top 14 countries, correcting for the overall change in output within each country. The relative change thus measures the extent to which AD research has increased compared with research in other disease areas within each country. France had the third largest relative increase in scientific output in AD between the periods 2004–2008 and 2009–2013 (1.7 times higher than the overall increase in scientific output across all disease areas), behind only India (2.5) and China (1.9). In both France and Spain, the relative increase in scientific output in AD was larger than any of the other four major comparator disease areas.

### Impact of French scientific research output in Alzheimer disease

Table 3 shows the relative change in the level (number of articles) and impact (proportion of articles in top 1 % globally cited) of French scientific research output in AD relative to the four major comparator disease areas between the periods 2004–2008 and 2009–2013. These results indicate that French scientific output in AD increased 3.9-fold more than the overall level of French scientific output between the periods 2004–2008 and 2009–2013, reflecting faster growth in AD research output than was observed for stroke, Parkinson disease, HIV, and diabetes. This quantitative rise also appeared to be reflected in an increase in the relative impact of articles in AD. There was a 2.3-fold increase between the periods 2004–2008 and 2009–2013 in the proportion of French AD articles in the top 1 % globally cited, which was also a greater rise than that observed for all of the four major comparator disease areas.

**Table 3:** Change in the impact of French Alzheimer disease research between the periods 2004–2008 and 2009–2013, as assessed by research output and representation in the top 1 % globally cited articles compared with French scientific output in stroke, Parkinson disease, HIV, and diabetes

	Research output (% France’s share of output)		Output relative increase (fold change)	France’s share of top 1 % globally cited articles		Top 1 % relative increase (fold change)
	2004–2008	2009–2013		2004–2008	2009–2013	
Alzheimer disease	1, 082 (0.4 %)	1, 836 (0.6 %)	3.9	18 (0.7 %)	44 (1.1 %)	2.3
Diabetes	2, 890 (1.1 %)	3, 725 (1.2 %)	1.6	75 (3.0 %)	130 (3.2 %)	1.2
HIV	3, 092 (1.1 %)	3, 297 (1.0 %)	0.4	43 (1.7 %)	58 (1.4 %)	0.6
Parkinson disease	997 (0.4 %)	1, 281 (0.4 %)	1.6	17 (0.7 %)	21 (0.5 %)	0.4
Stroke	1, 495 (0.5 %)	2, 264 (0.7 %)	2.9	46 (1.8 %)	98 (2.0 %)	1.8
Overall	270, 314	318, 946	1	2, 496	4, 041	1

Table 4 shows the output of AD articles, the relative citation impact, and the number and proportion of articles represented in the top 1 % cited globally for the six EU countries that contributed the most between 2004 and 2013. Whereas the United Kingdom and Germany contributed the greatest number of AD articles during both 2004–2008 and 2009–2013, the relative citation impact rose for all six countries, with an increase from 1.3 to 1.9 for France. Similarly, the proportion of top 1 % articles increased for all countries, including France (from 1.7 % to 2.4 %). Further analysis of the top 1 % articles showed that the predominant field of research in AD varied among countries. The proportion of articles on clinical investigation was >30 % in the United Kingdom, the Netherlands, Spain, and Italy and approximately 20 % in France and Germany. Compared with the other EU countries, French articles in AD showed a greater representation of research in epidemiology, genetics, and imaging.

**Table 4:** Relative impact of French Alzheimer disease research between the periods 2004–2008 and 2009–2013, as assessed by research output and representation in the top 1 % globally cited articles for France and the other top 5 leading EU countries

	2004–2008				2009–2013			
Country	Output of articles	Relative citation impact	Number in top 1 % globally cited articles	Percent in top 1 % globally cited articles	Output of articles	Relative citation impact	Number in top 1 % globally cited articles	Percent in top 1 % globally cited articles
France	1082	1.34	18	1.66	1836	1.92	44	2.40
Germany	1596	1.56	25	1.57	2316	2.16	65	2.81
Italy	1274	1.38	13	1.02	1797	1.67	30	1.67
Netherlands	720	1.78	17	2.36	1087	2.72	28	2.58
Spain	1001	1.02	4	0.40	1884	1.76	22	1.17
United Kingdom	1681	1.66	43	2.56	2294	2.59	81	3.53

## Discussion

Our results show an overall acceleration following the Third French Alzheimer Plan of the academic scientific output of France in AD research in terms of both volume and impact. Bibliometric analysis enabled assessment of the scientific impact of the plan on different geographical areas within France. The results show that six research areas contributed to almost 50 % of French AD articles. A positive correlation was observed between the level of financial support and global scientific contribution of the different geographical areas, although no correlation was detected between level of funding and article quality and/or impact. The increase in French AD research output was higher than for the four comparator disease areas and was the highest among other major European countries. Taken together, our bibliometric results show a specific impact of the Third French Alzheimer Plan.

Although the results of bibliometric approaches must be interpreted with caution and due consideration, given their limitations [4–6], the main points for discussion from our study are not a bibliometric ranking of research between different countries or different French geographical research areas per se, but the answer to two major questions: (1) Did the funding of a national Alzheimer plan expand academic research on AD and related dementias (if yes, where and by how much)? (2) What conclusions can be drawn for future consideration when this evidence is made public to encourage and increase the productivity of academic research (in which specific fields and by which collaborations)?

Despite the context of a global proliferation of scientific publications, which reflects as much an increase in production as it does many changes in editorial policies and pressures to publish, our study leaves no doubt that the French research contribution to AD increased considerably between the periods 2004–2008 and 2009–2013 in terms of both volume (number of articles) and impact (assessed by the relative representation of articles in the top 1 % globally cited). French scientific output in AD increased more in quantity and quality by comparison to several major countries and, within the country itself, by comparison with research productivity in other fields, such as Parkinson disease, HIV, and diabetes. It is noteworthy that with stroke, a field supported by a specific plan (2010–2014) [7], the difference is less marked. Our study showed that, at the national level, the CHUs are major players in French research, participating in 48 % of French AD publications. Almost all CHUs increased their output after the Third French Alzheimer Plan, improving both their SIGAPS score (based on journal impact factor and author position) and their proportion of top 10 % globally cited publications (with or without leadership position). Analyses by geographical area demonstrated notable heterogeneity at a regional level, with the majority of the scientific output coming from six areas. Whereas some of these contained centers that are well established in AD research, two small, focused research areas emerged as a result of the plan. We compared the percentage of top 10 % cited publications, rather than the number of top 10 % publications (which is related to the number of researchers), as a measure of impact. Whereas during the 2004–2008 period only four research areas had more than 20 % of their articles in the top 10 % most cited (Caen, Paris 06, Paris 07, and Bordeaux), after the plan 16 research areas reached that level and 4 were above 30 % (Angers, Toulouse, Rouen, and Dijon). It is noteworthy that all fields of biomedical research are not equally developed in all geographical research areas, and public knowledge about the diversity of the strongest points of each site may encourage collaborations in large-scale projects at a national level.

Our juxtaposition of the level of funding and production of original articles should be interpreted with caution. First, a public health plan is more than a financing issue. Through governmental support, such a plan should generate a nationwide mobilization to engage a range of stakeholders across scientific, medical, and social networks (e.g., the National Center for Ethics, France Alzheimer, and the Médéric Alzheimer Foundation). Fifty universities offered young researchers, pharmacists, and physicians 2-year hospital positions and a 1-year training grant on drug development, which led to recruitment of a total of 72 health professionals. The Third French Alzheimer Plan also mobilized broader activities across the EU at the time of French presidency in 2008. A bibliometric analysis focusing on scientific literature cannot measure the wider societal impact of such an investment, in France and in the EU in terms of raising awareness of AD. Second, there is a significant lag time between funding allocation and the publication of scientific articles. Although our review considered a 5-year time period after initiation of the plan, this may not be sufficient for areas such as clinical research, where there are significant administrative and ethical issues to be addressed in implementing new projects. Indeed, the choice was made in France not to create a new institute or agency similar to what was done for cancer and HIV research. Instead, with a very small administrative structure, the Third French Alzheimer Plan rapidly delivered support for previously productive areas of research (e.g., epidemiology and genetics) within 2 years. More comprehensive infrastructure projects, such as the Memento Cohort and the Acquisition Center and Image Processing, were then initiated, but they will deliver results over a longer time frame than 5 years.

With these caveats in mind, analysis of the correlation between level of funding and scientific output across French geographical research areas yielded some interesting results. Whereas there was a strong correlation between funding and the volume of scientific output, no such correlation was observed between funding and article impact (proportion of top 10 % articles). It may therefore be more appropriate for future allocation of research funding to take into account bibliometric analyses such as ours that assess the impact of a group’s research in addition to the volume of their output. Along similar lines for CHUs, the strong correlation observed between SIGAPS score and volume of scientific output was expected. The lack of correlation between SIGAPS score and the proportion of top 10 % cited articles strengthens arguments that assessments of research groups must take into account both the impact factor of target journals and publication citations to draw appropriate conclusions on performance. There may be multiple reasons for the observed lack of correlation between funding and article impact; for example, some areas of research were of greater current interest and were therefore more likely to attract more citations during the period of evaluation (e.g., neuroimaging in the Caen area). Similarly, some research projects are more internationalized and may therefore attract more citations (e.g., clinical investigations in Toulouse). Some geographical research areas are likely to have benefited from other, international sources of funding to conduct high-impact research and could therefore have generated publications with a scientific output apparently uncorrelated with the level of funding from the Third French Alzheimer Plan.

At the global level, the United States is the leading country for scientific research. It produced almost 26,000 articles in AD (approximately 40 % of global output in the field) during the period 2004–2013. The majority of funding for AD research in the United States comes from the federal government via the National Institute on Aging (NIA), and, since 1985, AD Centers and AD research Centers have been established in major medical schools. In France, by contrast, university memory centers for research and appeal were only established in 2002, and only during the Third French Alzheimer Plan were these extended nationwide. Despite the 2008 financial crisis, major investments in Alzheimer research were announced by several countries in Europe (notably the United Kingdom and Germany) and, later on, by the United States. According to our analysis, the United Kingdom and Germany remain major contributors in AD, ahead of France. Several other countries also contributed in quantity and quality to worldwide academic output, in some cases (Australia, the Netherlands, and Germany) as a result of national AD/dementia plans. There are as yet no national dementia plans in other countries, such as China, India, and South Korea [8–11], but these countries face a major demographic challenge in terms of the age profile of their populations, and it is notable that they have dramatically accelerated their research in AD and other disease areas. Following this first attempt to identify an association between a national investment in research on a disease and the level of scientific output, it would now be useful to investigate in these other countries (1) the final grant attributions by comparison with the initial political announcements and (2) the evolution in each country’s global scientific output. An international working group on this issue would be helpful to improve and standardize methodology for the selected indices and address the delays of follow-up between grant attribution and publication of original articles.

## Conclusions

Our study provides evidence of the probable impact of a national effort in France to stimulate improved diagnosis and treatment of AD and dementia, which focused on original research as far as possible in the economic context. We believe that bibliometric assessment and analysis, whatever its limits, should be mandatory at a time when many countries have launched specific initiatives to promote research in AD and dementia (exemplified by the recent Global Action Against Dementia summit). Given that a minimum 5-year interval is essential to ensuring a sufficient production of original articles and to measure their impact, we advocate a continuous process for bibliometric assessment rather than a one-time, snapshot approach. Such an approach would contribute to better orientation of scientific policies within and between countries and encourage national and international collaborations around the most productive sites in certain fields of research, rather than using bibliometric analysis primarily as a tool to benchmark universities or countries.

## Additional file

Additional file 1:
**Figure showing the distribution of the funding of the French Alzheimer Plan and the distribution by research field.** **Figure S1. a** Research funding of the French Alzheimer Plan by program. ^a^Genome-wide association study (GWAS): an ongoing program of genetic research based on previously collected and documented DNA samples. ^b^Three Cities (3C) Study: a population cohort research study. ^c^National Platform for Image Acquisition and Processing (CATI): a nationwide infrastructure program. ^d^A Methodological Research Group to manage a new cohort of 2300 patients in the prodromal stage of Alzheimer disease attending university memory clinics (the Memento Cohort): a nationwide infrastructure program. ^e^Included 50 supplementary hospital positions for 2 years, competitively allocated to young scientists, pharmacists, and physicians, and a 1-year training program on international development of Alzheimer drugs, which attracted 72 young and senior health professionals. ^f^Center for Early Onset Alzheimer Disease (CNRMAJ): establishment of a network around Rouen, Lille, and Paris to recruit 225 families fulfilling the criteria of early-onset dementia (before age 65 years) and one confirmed case of Alzheimer disease in the family. ^g^National Data Bank for Alzheimer Disease patients (Nice CM2R): creation of a program to collect standardized online information nationwide from all patients referred to memory clinics or university memory clinics from 2009 onward. In 2012 and 2013, a total of 177,242 new patients were added to the National Data Bank. **b** Research funding of the French Alzheimer Plan by program and by research field. Field of research was defined according to the title and abstract of the grant proposals. The Ministry of Health supported 50 projects of clinical research through Hospital Programs for Clinical Research (PHRCs). Public research organizations (RPO) supported by the French National Research Agency (ANR) and the Alzheimer Plan Foundation. CHU, university hospitals. (PDF 214 kb)

## Abbreviations

AD: Alzheimer disease and related disorders
ANR: National Agency for Research
CADASIL: cerebral autosomal dominant arteriopathy with subcortical infarcts and leukoencephalopathy
CHU: university hospital
ESI: Thomson Reuters Essential Science Indicators
EU: European Union
PHRC: Hospital Program for Clinical Research
RPO: Public Research Organization
