# Supplementary material for: The Third French Alzheimer Plan: analysis of the influence of a national public health initiative on scientific research productivity and impact
Source: Alzheimers Res Ther. 2015 Sep 26;7:60. doi: 10.1186/s13195-015-0144-z (PMC4584022; doi:10.1186/s13195-015-0144-z)
Supplement: Additional file 1: — Figure showing the distribution of the funding of the French Alzheimer Plan and the distribution by research field. Figure S1. a Research funding of the French Alzheimer Plan by program. aGenome-wide association study (GWAS): an ongoing program of genetic research based on previously collected and documented DNA samples. bThree Cities (3C) Study: a population cohort research study. cNational Platform for Image Acquisition and Processing (CATI): a nationwide infrastructure program. dA Methodological Research Group to manage a new cohort of 2300 patients in the prodromal stage of Alzheimer disease attending university memory clinics (the Memento Cohort): a nationwide infrastructure program. eIncluded 50 supplementary hospital positions for 2 years, competitively allocated to young scientists, pharmacists, and physicians, and a 1-year training program on international development of Alzheimer drugs, which attracted 72 young and senior health professionals. fCenter for Early Onset Alzheimer Disease (CNRMAJ): establishment of a network around Rouen, Lille, and Paris to recruit 225 families fulfilling the criteria of early-onset dementia (before age 65 years) and one confirmed case of Alzheimer disease in the family. gNational Data Bank for Alzheimer Disease patients (Nice CM2R): creation of a program to collect standardized online information nationwide from all patients referred to memory clinics or university memory clinics from 2009 onward. In 2012 and 2013, a total of 177,242 new patients were added to the National Data Bank. b Research funding of the French Alzheimer Plan by program and by research field. Field of research was defined according to the title and abstract of the grant proposals. The Ministry of Health supported 50 projects of clinical research through Hospital Programs for Clinical Research (PHRCs). Public research organizations (RPO) supported by the French National Research Agency (ANR) and the Alzheimer Plan Foundation. CHU, university hospitals. ( [file 13195_2015_144_MOESM1_ESM.pdf]

Funding research (€ 000s)	Total	*CHU***	*RPOs****
PHRC projects	24 686	*24 686*	
ANR projects	34 107	*2 196*	*31 911*
Foundation programmes:			
- *Project applications*	*8 481*	*1 740*	*6 741*
- *Post-doctoral positions*	*3 266*		*3 266*
- *GWAS*[a]	*3 997*		*3 997*
- *Three cities study*[b]	*2 298*		*2 298*
- *CATI*[c]	*9 000*		*9 000*
- *Memento cohort*[d]	*14 828*	*10 730*	*4 098*
Total foundation:	41 870	*12470*	*29400*
Training and education[e]	15 412		
Centre for early onset Alzheimer Disease (CNRMAJ)[f]	3 000	*3 000*	
National data bank for Alzheimer Disease patients (Nice CM2R)[g]	6 504	*6 504*	
Global funding	125 579	48 856	61 311
Total	101 751		

Figure 1(a)

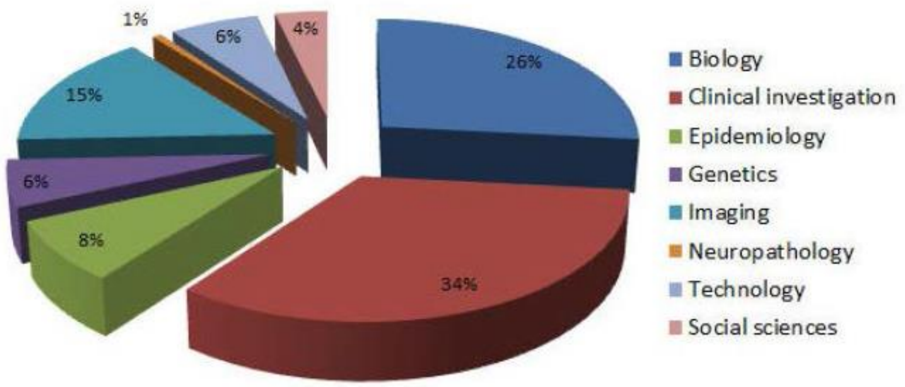

Figure 1(b)
